# What the Hel: recent advances in understanding rifampicin resistance in bacteria

**DOI:** 10.1093/femsre/fuac051

**Published:** 2022-12-22

**Authors:** Petra Sudzinová, Hana Šanderová, Tomáš Koval', Tereza Skálová, Nabajyoti Borah, Jarmila Hnilicová, Tomáš Kouba, Jan Dohnálek, Libor Krásný

**Affiliations:** Laboratory of Microbial Genetics and Gene Expression, Institute of Microbiology of the Czech Academy of Sciences, Vídeňská 1083, 14220 Prague, Czech Republic; Laboratory of Microbial Genetics and Gene Expression, Institute of Microbiology of the Czech Academy of Sciences, Vídeňská 1083, 14220 Prague, Czech Republic; Laboratory of Structure and Function of Biomolecules, Institute of Biotechnology of the Czech Academy of Sciences, Centre BIOCEV, Průmyslová 595, 25250 Vestec, Czech Republic; Laboratory of Structure and Function of Biomolecules, Institute of Biotechnology of the Czech Academy of Sciences, Centre BIOCEV, Průmyslová 595, 25250 Vestec, Czech Republic; Laboratory of Microbial Genetics and Gene Expression, Institute of Microbiology of the Czech Academy of Sciences, Vídeňská 1083, 14220 Prague, Czech Republic; Laboratory of Microbial Genetics and Gene Expression, Institute of Microbiology of the Czech Academy of Sciences, Vídeňská 1083, 14220 Prague, Czech Republic; Cryogenic Electron Microscopy Research-Service Group, Institute of Organic Chemistry and Biochemistry of the Czech Academy of Sciences, Flemingovo náměstí 2, 16000 Prague, Czech Republic; Laboratory of Structure and Function of Biomolecules, Institute of Biotechnology of the Czech Academy of Sciences, Centre BIOCEV, Průmyslová 595, 25250 Vestec, Czech Republic; Laboratory of Microbial Genetics and Gene Expression, Institute of Microbiology of the Czech Academy of Sciences, Vídeňská 1083, 14220 Prague, Czech Republic

**Keywords:** rifampicin, antibiotics, resistance, bacteria, RNA polymerase, HelD/HelR

## Abstract

Rifampicin is a clinically important antibiotic that binds to, and blocks the DNA/RNA channel of bacterial RNA polymerase (RNAP). Stalled, nonfunctional RNAPs can be removed from DNA by HelD proteins; this is important for maintenance of genome integrity. Recently, it was reported that HelD proteins from high G+C *Actinobacteria*, called HelR, are able to dissociate rifampicin-stalled RNAPs from DNA and provide rifampicin resistance. This is achieved by the ability of HelR proteins to dissociate rifampicin from RNAP. The HelR-mediated mechanism of rifampicin resistance is discussed here, and the roles of HelD/HelR in the transcriptional cycle are outlined. Moreover, the possibility that the structurally similar HelD proteins from low G+C *Firmicutes* may be also involved in rifampicin resistance is explored. Finally, the discovery of the involvement of HelR in rifampicin resistance provides a blueprint for analogous studies to reveal novel mechanisms of bacterial antibiotic resistance.

## Teaser

Interactions of bacterial HelD/HelR proteins with RNA polymerase, their involvement in rifampicin resistance & expression control by rifampicin are presented and discussed.

## Introduction

Unlike in experimental settings in the laboratory involving single species, bacteria exist in Nature in communities. Within these communities, all types of interactions can be found, from symbiosis to indifference to open hostility. To survive and navigate this complex environment, bacteria must sense, interpret, and respond to numerous signals and protect themselves against various toxic compounds, such as antibiotics.

Many of the currently used antibiotics were discovered in *Actinobacteria* (Mast and Stegmann 2019), a phylum of mostly gram-positive bacteria of great medicinal and economical importance. Rifampicin (synonym rifampin) was discovered in the mid-1960’s, and was soon introduced into therapeutic use (Sensi 1983). Rifampicin (and its derivatives) functions by binding to the β subunit (encoded by the *rpoB* gene) of bacterial RNA polymerase (RNAP) in its DNA/RNA channel. When bound, rifampicin obstructs this channel and prevents extension of RNA during transcription initiation beyond the first 2–3 nucleotides (nt), effectively shutting down transcription, the first step of gene expression (Campbell et al. 2001, Lin et al. 2017). Conversely, when RNA is longer than 3 nt, it blocks the rifampicin binding site.

Resistance to rifampicin arises due to mutations in amino acids (aa) involved in its binding to RNAP (Leehan and Nicholson 2021). These mutations adversely affect the activity of RNAP and this is counteracted by secondary mutations in RNAP that restore fitness (Kurepina et al. 2022). In many *Nocardia* species there are even two copies of *rpoB*, one copy (*rpoB2*) dedicated to providing resistance to rifampicin (Ishikawa et al. 2006). Moreover, bacteria have developed several additional approaches to deal with rifampicin, either by active efflux (Li et al. 2015, Machado et al. 2018) or by modifications such as phosphorylation, ADP-ribosylation, glycosylation, or hydroxylation (Tupin et al. 2010, Goldstein 2014, Stogios et al. 2016). These modifications alter the binding properties of rifampicin, preventing its stable interaction with RNAP. Recently, a novel mechanism of bacterial resistance to rifampicin was reported, mediated by the HelD protein in *Actinobacteria* (Hurst-Hess et al. 2022, Surette et al. 2022). The authors proposed to refer to this protein as HelR in this phylum. In this review, the term ‘HelD’ is used for both HelD and HelR, regardless of the species, unless specified otherwise. This review addresses structural and functional aspects of the mechanism of action of HelD/HelR proteins and discusses the so far poorly characterized mechanisms of their expression control.

## HelD—discovery & functional interaction with RNAP

HelD is a helicase-like ATPase/GTPase without a helicase function. It was first investigated with respect to homologous recombination in *Bacillus subtilis* (*Bsu*) (Carrasco et al. 2001). A decade later, it was detected as an RNAP associating protein in *B. subtilis* (Delumeau et al. 2011). Subsequent *in vitro* studies then established that HelD dissociates stalled/inactive complexes of RNAP from DNA, liberating the template for further transcription and preventing potentially deleterious transcription-replication collisions, thereby contributing to genome integrity maintenance (Wiedermannova et al. 2014). This function of HelD is synergistically enhanced by δ, a small subunit of RNAP specific for *Firmicutes* (Juang and Helmann 1994, Doherty et al. 2010, Motackova et al. 2010, Rabatinova et al. 2013, Kuban et al. 2019), a phylum where *B. subtilis* belongs. HelD thus represents one of several mechanisms that ensure removal of RNAPs roadblocking DNA (Wiedermannova and Krasny 2021). This functional redundancy is probably reflected in the minor phenotypes of the absence of HelD—slower growth in the presence of elevated KCl concentration and prolonged lag phase after dilution of stationary phase cells into fresh medium (Wiedermannova et al. 2014). The latter phenotype is also consistent with another proposed function for HelD—storing inactive RNAPs in stationary phase (Pei et al. 2020). The sequestered RNAPs are reactivated when the cells encounter more advantageous conditions, reminiscent of similar roles of 6S and Ms1 sRNAs that sequester the primary σ factor-containing RNAP holoenzyme or the RNAP core (subunit composition α_2_ββ’ω), respectively (Wassarman 2018, Vankova Hausnerova et al. 2022).

## HelD—structural aspects

**Figure 1.**
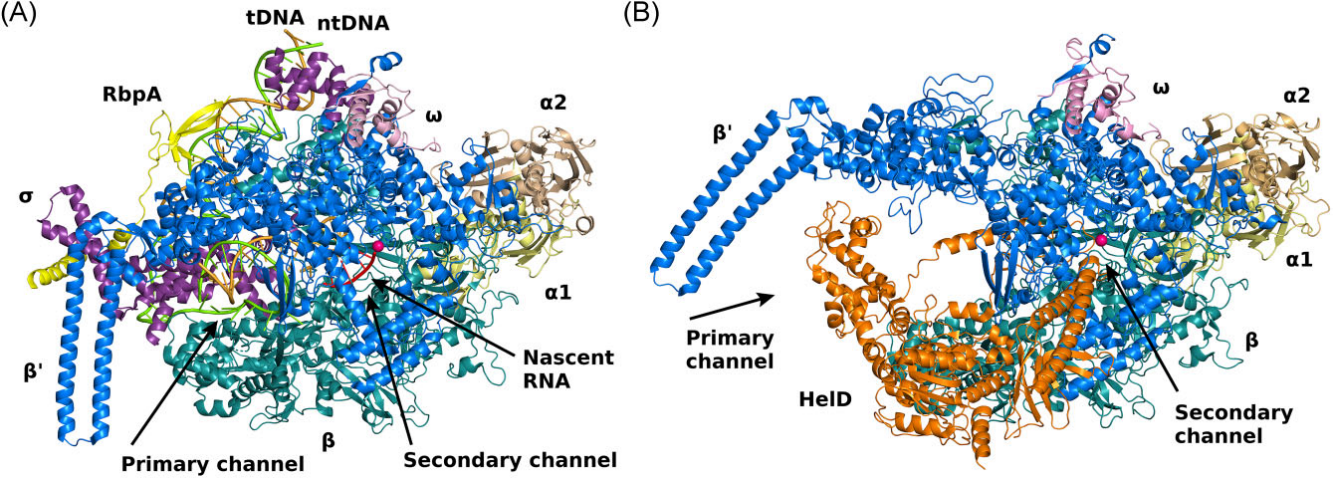
*Msm* RNAP and its interaction with nucleic acids (A) or HelD (B). (**A**) Initiation complex of *Msm* RNAP (PDB id 5VI5, Hubin et al. 2017) in secondary structure representation, color coding: RNAP α1, pale yellow; α2, wheat; β, teal; β’, blue; ω, pink; σ, dark purple; RbpA, bright yellow; template DNA strand, bright orange; non-template DNA strand, bright green; nascent RNA, red; the active site of RNAP is marked with modeled Mg^2+^—hotpink sphere. (**B**) Complex of the *Msm* RNAP core with HelD (PDB id 6YYS, Kouba et al. 2020) in secondary structure representation, color coding as in (A) with the addition of HelD in orange; Mg^2+^ experimentally localized in the active site—hotpink sphere. The graphics were created using PyMOL (The PyMOL Molecular Graphics System, Version 2.0, Schrödinger, LLC).

In 2020, the structure of HelD was determined in complex with RNAP by cryo-EM. Three structural studies of HelD and RNAP were published side-by-side at that time, two from *B. subtilis* and one from *Mycobacterium smegmatis* (*Msm*) (Kouba et al. 2020, Newing et al. 2020, Pei et al. 2020). These studies revealed a unique type of interaction between RNAP and a protein factor. HelD from both species penetrates both the primary channel (where DNA binds) and secondary channel (where NTPs enter the active site), embracing RNAP. This causes large conformational changes in RNAP. The claws of the two largest subunits, β and β’, that form the primary channel become wide open, which, in turn, together with the obstructing presence of HelD on RNAP and widening of the RNA exit channel, is incompatible with the presence of nucleic acids (Fig. 1).

**Figure 2.**
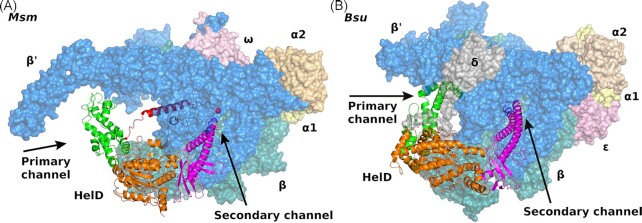
Comparison of *Msm* and *B*s*u* HelD proteins and their binding to respective RNAP core enzymes. The views correspond to superposition based on β domains. **(A)***Msm* RNAP-HelD complex (PDB id 6YYS, Kouba et al. 2020), color coding: RNAP as in Fig. 1, HelD: N-terminal domain, magenta; ATPase domains, orange; CO domain, green; PCh loop, red. RNAP is shown as molecular surface and spheres, HelD in secondary structure representation. (**B)***Bsu* RNAP-HelD complex (PDB id 6ZFB, Pei et al. 2020), color coding: RNAP—α1, yellow; α2, wheat; β, teal; β’; blue; ε, dark pink; δ, grey; HelD—N-terminal domain, magenta; ATPase domain, orange; CO domain, green. The graphics were created using PyMOL.

HelD proteins from both organisms, *Bsu and Msm*, contain two ATPase RecA-like domains and the Walker motif, which indicates ATP/GTP binding (Walker et al. 1982). Indeed, HelD proteins bind and hydrolyze ATP and/or GTP, which leads to conformational changes in HelD (Koval et al. 2019). Nevertheless, it is not clear at which step the hydrolysis occurs and the details of these conformational changes are not defined yet. In all available RNAP-HelD complexes, HelD is present without a nucleoside triphosphate (NTP). Addition of ATP or its nonhydrolyzable analogue then induces dissociation of HelD from RNAP in *B. subtilis* (Pei et al. 2020), arguing that the changes required for HelD release are caused by binding but not hydrolysis of ATP. HelDs from both organisms also possess the clamp opening (CO) domain that interacts with β’, holding the DNA clamp wide open, and the N-terminal domain that inserts into the secondary channel of RNAP (Fig. 2).

**Figure 3.**
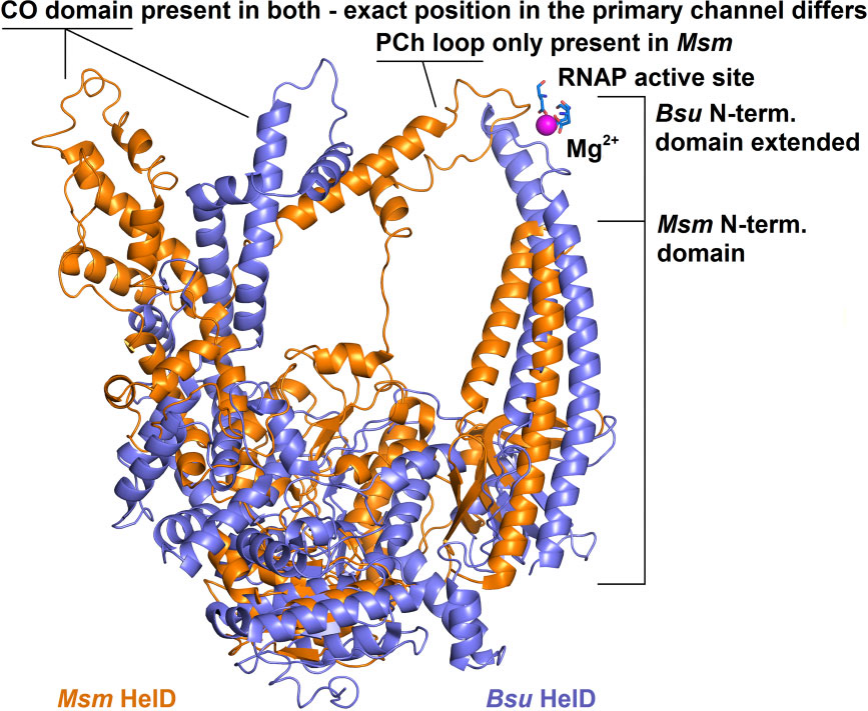
Comparison of *Msm* and *Bsu* HelD structures. The molecules are superimposed as in Fig. 2, *i.e*. in equivalent positions when binding RNAP. Both molecules are in secondary structure representation. *Msm* HelD is colored orange, *Bsu* HelD purple. Mg^2+^ present in the active site of *Msm* RNAP is shown as magenta sphere. Amino acids of the active site coordinating it are shown as sticks with carbon in blue. The most profound differences between HelD from *Msm* and *Bsu* are marked. The graphics was created using PyMOL.

HelDs from the two organisms, however, differ in some aspects of their structures (Fig. 3), and, consequently in their interaction with RNAP. In the case of *Bsu* HelD, the N-terminal domain reaches deep into the secondary channel, interfering with the active site of RNAP. In the case of *Msm* HelD, the N-terminal domain does not reach as deep but still immobilizes the trigger loop (TL) as in *Bsu* HelD (Newing et al. 2020, Pei et al. 2020). The TL is a flexible component of RNAP that cycles between ‘open’ and ‘closed’ states with respect to nucleoside triphosphate (NTP) entry to the active site (Mazumder et al. 2020). The interaction of the N-terminal domain of HelD with the TL freezes the TL in an open-like state, which interferes with the NTP addition cycle (Kouba et al. 2020). *Bsu* HelD then lacks the primary channel (PCh) loop by which *Msm* HelD reaches from inside the primary channel directly to the active site.

The structural differences are also reflected in terminology: *Bsu*-like HelDs are termed Class I and *Msm*-like HelDs Class II (Larsen et al. 2021). Class I HelDs are found in the low G+C Gram-positive bacteria and gram-negative *Bacteroidia*, in the latter case likely acquired by horizontal gene transfer from anaerobic gut *Clostridiales* due to environmental coexistence of these organisms. Class II HelDs are present in the high G+C Gram-positive bacteria where some genera, such as *Streptomyces, Nonomuraea*, and *Frankia*, even contain several Class II HelD paralogs (Larsen et al. 2021). Figure 3 illustrates the prominent structural differences between HelD proteins from both classes. Very recently, Class III HelD proteins were identified bioinformatically by Larsen *et al*. in Gram-negative Deltaproteobacteria (Larsen et al. 2021). A hallmark of Class III HelDs is an extended motif within the N-terminal domain, DWRX[A/S]P (extended by one aa, X), and the absence of the PCh loop. Furthermore, their N-terminal domain, expected to bind into the secondary channel of RNAP, is shorter than this domain in Class I HelDs and longer than in Class II HelDs. Examples of bacterial species with identified *helD* genes (Class I, Class II, and Class III) are in Table 1.

**Table 1.** Classes of HelD proteins with representative bacterial species. Class I are low G+C Gram-positive bacteria and Gram-negative *Bacteroidia*, Class II are high G+C Gram-positive bacteria. Class III are found in Gram-negative bacteria. For a more detailed list and phylogeny see (Larsen et al. 2021).

HelD Class	Group	Species	Gene name
**I**	*Bacilli*	*Bacillus subtilis* 168	BSU 33450 (*yvgS, helD*)
		*Bacillus cereus* AH187	BCAH187_A1206, BCAH187_A2861
		*Bacillus anthracis* AMES	BA_1040, BA_2814
		*Lactobacillus acidophilus* NCFM	lac_1676
		*Enterococcus faecium* Aus0004	EFAU004_01304, EFAU004_00387
		*Staphylococcus delphini* NCTC12225	sdp_01978
	*Clostridia*	*Clostridioides difficile* 630	CD630_04550
		*Clostridium botulinum A* ATCC19377	CLB_2867, CLB_3399
	*Bacteroidia*	*Bacteroides thetaiotaomicron* VPI-5482	BT_1890
**II**	*Acidimicrobiia*	*Ilumatobacter coccineus*	aym_09360, aym_20540
	*Actinobacteria*	*Mycobacterium smegmatis* mc^2^ 155	MSMEG_2174
		*Mycobacterium abscessus* ATCC 19977	MAB_3189c (*helR*)
		*Mycobacterium tuberculosis microti* OV254^1^	PLV44927.1 (*helD*)
		*Rhodococcus equi* 103S	REQ_25070, REQ_15310
		*Nocardia asteroides* NCTC11293	nad_03000, nad_04408
		*Bifodobacterium bifidum* PRL2010	bbp_0546
		*Brevibacterium flavum* ZL-1	bfv_07580
		*Corynebacterium glutamicum* ATCC13031	CG_1555
		*Corynebacterium diptheriae* NTCC13129	DIP_1156
		*Streptomyces venezuelae*	SVEN_6029 (*helRSv, helD*), SVEN_5092, SVEN_2719, SVEN_4127, SVEN_3939
		*Streptomyces coelicolor* A3(2)	SCO5439, SCO2952, SCO4316, SCO4195
**III**	*Deltaproteobacteria*	*Myxococcus xanthus* DK 1622	MXAN_5482
		*Sorangium cellulosum* So157-2	SCE1572_03860

1 Other M. *tuberculosis* strains appear to lack HelD homologs.

## HelD—involvement in rifampicin resistance—discovery

Already in 2004, Hutter *et al*. reported that the *Bsu yvgS* (synonym for *helD*; it was a gene of unknown function then) promoter region was inducible by rifampicin (Hutter et al. 2004). A follow-up study confirmed this result and showed the inducibility of the *yvgS* promoter region also by another RNAP-targeting compound, streptovaricin (Urban et al. 2007). In 2017, Giddey *et al*. reported the HelD protein as one of the most upregulated proteins after sub-minimal inhibitory concentration (sub-MIC) rifampicin treatment in *M. smegmatis* (Giddey et al. 2017). In 2019, RNA isolated from *M. smegmatis* after exposure to sublethal doses of rifampicin was analyzed by RNA-seq and *helD* was one of the most induced genes (Hurst-Hess et al. 2019). Very recently, two excellent studies reported convergent results of the involvement of HelD in rifampicin resistance, using two different actinobacterial species: *Mycobacterium abscessus* and *Streptomyces venezuelae* (Hurst-Hess et al. 2022, Surette et al. 2022).

*Mycobacterium abscessus* is a distant relative of *Mycobacterium tuberculosis* and *Mycobacterium leprae* but in comparison to these pathogens it is a fast-growing bacterium (Maurer et al. 2014). *M. abscessus* can cause infections in humans. These infections are usually limited to the skin and the soft tissues under the skin, although in persons with various chronic lung diseases, such as cystic fibrosis it can also cause serious lung infections. Notably, *M. abscessus* is known for its resistance to rifampicin, unlike *M. tuberculosis* and *M. leprae* that do not usually contain the *helD* gene (see Table 1). This absence may be due to the environment that these human pathogens inhabit, where they have not been exposed to these antibiotics as much over a long evolutionary time frame. Conversely, *S. venezuelae* is a soil-dwelling organism where it faces the challenge from antibiotic producers (Chater 2016). Such organisms typically possess multiple lines of defense against antibiotics to enhance their chances of survival in the hostile environment.

Hurst-Hess *et al*. treated *M. abscessus* with sub-MIC of rifampicin and helD mRNA (MAB_3189c gene) was one of the two most upregulated mRNAs (Hurst-Hess et al. 2022). The other was arr (MAB *arr* gene), which is translated into ADP-ribosyltransferase that modifies and inactivates rifampicin (Morgado et al. 2021). Surette *et al*. used the previously identified rifampicin associated element (RAE) in the 5’ untranslated region (5’ UTR) of another rifampicin modifying enzyme—glycosyltransferase (*rgt*) to search for other genes linked to this sequence (Spanogiannopoulos et al. 2014). In *S. venezuelae*, this yielded two hits—rifamycin monooxygenase (*rox*) and HelD (HelR, SVEN_6029 gene) (Surette et al. 2022).

## Mechanisms of HelD-mediated rifampicin resistance

Both recent studies (Hurst-Hess et al. 2022, Surette et al. 2022) showed that deletion of the HelD-encoding gene results in increased sensitivity to rifampicin in the tested actinobacterial species. This correlates with the ability of HelD to dissociate stalled RNAPs (Kouba et al. 2020). Stalled RNAPs are also those bound to the promoter, arrested by rifampicin. When the rifampicin concentration is not saturating and HelD dissociates rifampicin-arrested RNAPs from promoters, rifampicin-free RNAP molecules can initiate transcription from these promoters. Indeed, Hurst-Hess *et al*. showed that HelD can do exactly that (Hurst-Hess et al. 2022). Although this may already seem to provide significant protection against rifampicin, Class II HelDs can do even better.

Using a rifampicin photoaffinity probe (RPP) linked to biotin, Surette *et al*. showed that *S. venezuelae* HelD decreases binding of RPP to RNAP, and, importantly, it can also dissociate rifampicin from RNAP (Surette et al. 2022). This dissociation seems to depend on the HelD PCh loop, and mutations of two amino acid residues at the tip of this loop to alanines (Glu496 and Asp497, numbering according to *M. abscessus*; Glu484 and Asp485 in *Msm* HelD, MSMEG_2174 gene) compromise the ability of HelD to render the cell rifampicin-resistant even though this HelD mutant is still capable of binding to RNAP, and dissociating stalled RNAPs from DNA (Hurst-Hess et al. 2022). This also suggests that dissociation of rifampicin from RNAP by HelD and not dissociation of the RNAP-rifampicin complex from DNA is the main mode of how HelD provides resistance to rifampicin.

**Figure 4.**
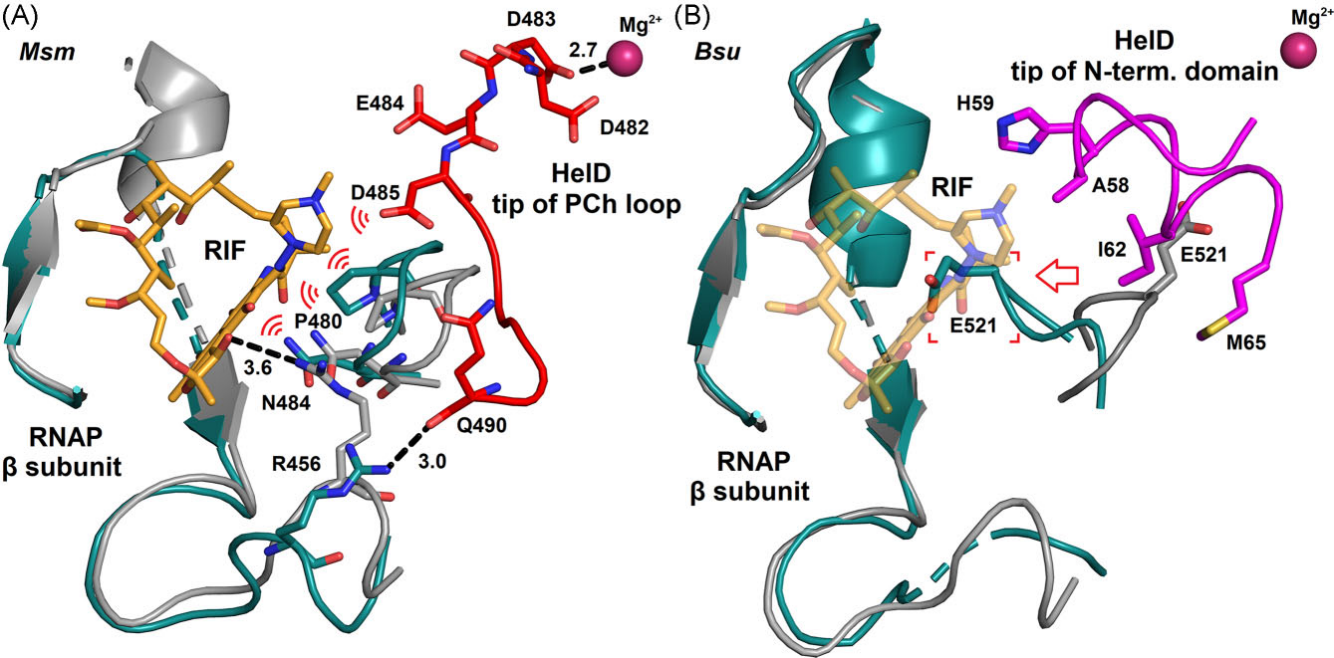
HelD causes conformational changes in the RNAP rifampicin binding site. (**A**) Structural comparison of the rifampicin binding site of the *Mtu* RNAP-rifampicin complex (PDB id 5UHC, Lin et al. 2017) with the same site from the *Msm* RNAP-HelD complex (PDB id 6YYS, Kouba et al. 2020) is shown. Binding of the *Msm* HelD PCh loop (shown as sticks with carbon in red and in red secondary structure representation) in the RNAP primary channel and active site deforms the rifampicin (RIF) binding pocket. Rifampicin is shown with carbon colored pale orange. Rifampicin binding pockets from both structures are shown in secondary structure representation (*Mtu* colored pale gray, *Msm* colored teal) with the selected residues shown as sticks. The indicated hydrogen bonds and Mg^2+^ coordination distances are given in Å. Possible atomic clashes between rifampicin and its deformed binding site and HelD are hinted with the red ‘wave’ symbols. (**B**) Structural comparison of expected rifampicin binding in *Bsu* RNAP (PDB id 6WVJ, Newing et al. 2020) and the same site in the complex of *Bsu* RNAP with HelD (PDB id 6ZFB (Pei et al. 2020)) superimposed by the main chain of β. Binding of the *Bsu* HelD N-terminal domain (shown as sticks and coil with carbon in magenta) in the RNAP secondary channel and active site (indicated with Mg^2+^) deforms the expected rifampicin binding pocket, which is indicated with the arrow; aa residues closest to the rifampicin pocket are shown as sticks. Rifampicin is shown as in panel A with increased transparency for clarity. The *Bsu* RNAP chains are shown in secondary structure representation (*Bsu* elongation complex colored pale gray, HelD complex colored teal) with selected aa residues shown as sticks. Potential atomic clashes between rifampicin and its deformed binding site are indicated with the red square brackets. The graphics was created using PyMOL.

A comparison of the *Msm* RNAP-HelD complex with the *Mycobacterium tuberculosis* (*Mtu*) RNAP-rifampicin complex reveals that the *Msm* HelD PCh loop affects the geometry of the rifampicin binding pocket (Fig. 4A). The PCh loop tip is within 3.5 Å of the RNAP rifampicin binding site and Asp485 can directly interact with the rifampicin binding pocket. Moreover, aa residues of the PCh loop that follow Asp485 in the peptide chain deform the pocket so that the presence of both—rifampicin and HelD—at the same time would likely lead to serious atomic clashes. Furthermore, Arg456 of the *Msm* β subunit (Arg461 in *M. abscessus*), corresponding to Arg465 in the *Mtu* RNAP-rifampicin complex (Lin et al. 2017), which forms a direct hydrogen bond to the antibiotic, is diverted from the rifampicin binding pocket in the *Msm* RNAP-HelD complex and forms a hydrogen bond to Gln490 of *Msm* HelD. This altered geometry of the rifampicin pocket likely leads to a decreased affinity for rifampicin binding. Additionally, ATP binding/hydrolysis to/by HelD may elicit additional conformational changes in the rifampicin pocket, promoting dissociation of the antibiotic from RNAP. Based on the relatively small extent of the conformational changes induced in the rifampicin binding pocket by HelD binding, it is unlikely that these changes are sufficiently long-lived to provide additional protection after HelD dissociation. Such a phenomenon was proposed for tetracycline ribosomal protection proteins (Wilson et al. 2020).

Mutants in the Walker motif of *M. abscessus* HelD were also defective in eliciting rifampicin resistance but they seemed to have a significantly decreased ability to bind RNAP, probably due to a compromised conformation (Hurst-Hess et al. 2022). Taken together, this classifies Class II HelD proteins as Type II target protection, an increasingly more appreciated mechanism of antibiotic resistance, where the target protection proteins induce conformational changes in the target that allosterically dissociate the antibiotic from the target (for review see (Wilson et al. 2020)).

The previous paragraphs of this section describe studies in high G+C *Actinobacteria* (Class II HelDs or HelRs). Surette *et al*. also tested the effect of the absence of a Class I HelD from *B. subtilis* on rifampicin MIC and detected no difference compared to wt (Surette et al. 2022). They concluded that Class I HelD proteins are likely not involved in rifampicin resistance. For Class III HelDs, no experimental evidence exists about their potential involvement in rifampicin resistance.

## Mechanisms of rifampicin-induced expression of HelD

Induction of transcription in bacteria by sub-MIC rifampicin has been reported previously (Yim et al. 2013), perhaps most notably for *rpoBC* genes (Howe et al. 1982, Zhu et al. 2018), encoding the two largest subunits of RNAP, β and β’. This induction was associated with increased resistance to rifampicin. However, upregulation of rpoBC mRNA in *M. smegmatis* (2x↑) was not as pronounced as upregulation of helD mRNA in *M. abscessus* (25x↑) (Zhu et al. 2018, Hurst-Hess et al. 2022). Nevertheless, it was shown that the *Msm rpoBC* operon is transcribed from two promoters, the first (more upstream relative to the genes) promoter is less active and interferes with transcription from the second promoter. When sub-MIC rifampicin is present, it preferentially inhibits transcription from the first promoter and this increases transcription from the second promoter (Zhu et al. 2018). This implies that the two promoters are differentially susceptible to binding of RNAP with/without rifampicin, a phenomenon that will be of interest to define in detail.

**Figure 5.**
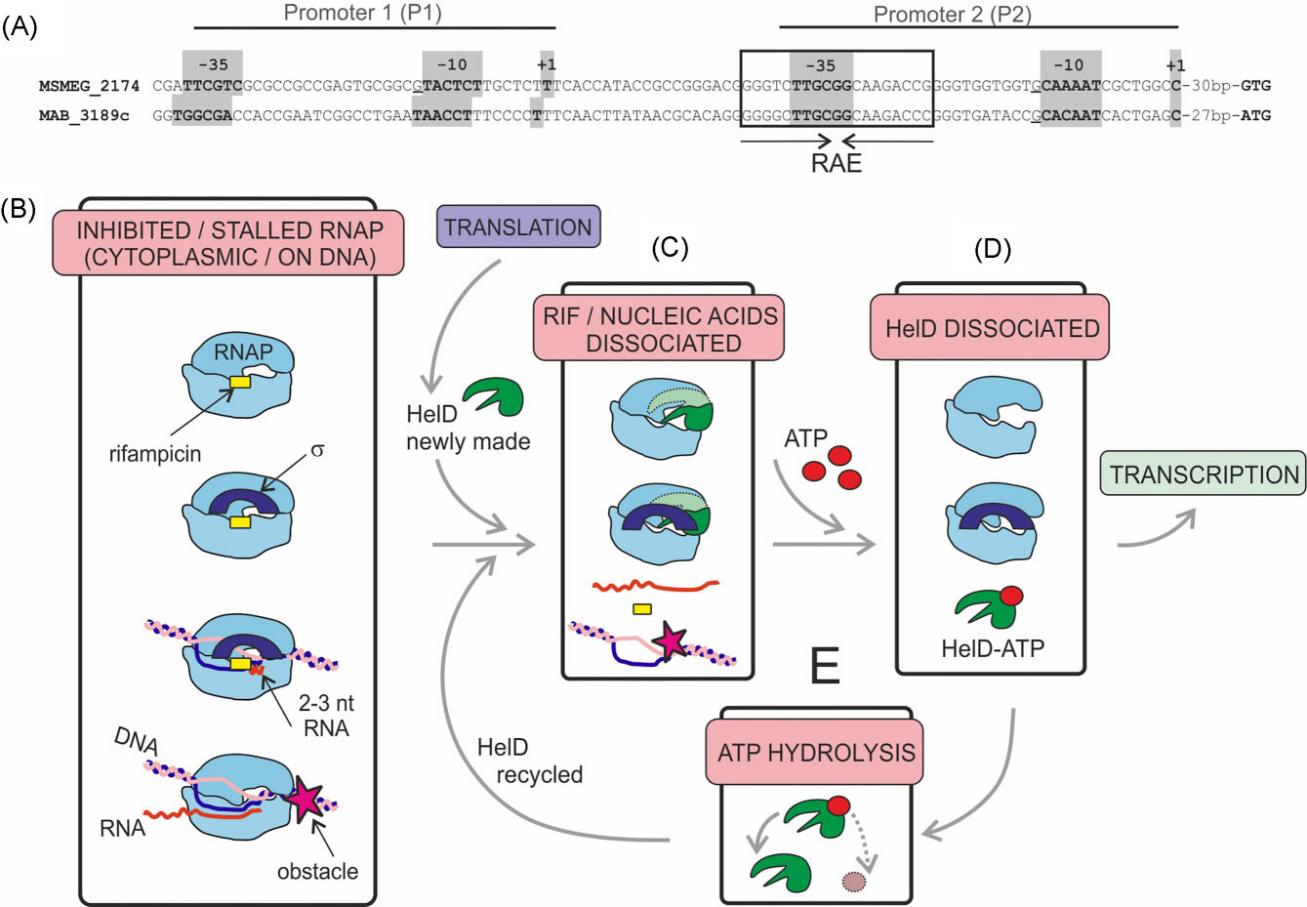
Class II HelD promoter region architecture and a model of the Class II HelD cycle. (**A**) Alignment of promoter sequences for *helD* gene in *M. smegmatis* (MSMEG_2174) and *M. abscessus* (MAB_3189c). The transcription start site (TSS) for P1 and P2 of *M. smegmatis* were experimentally determined (Martini et al. 2019); for *M. abscessus* the TSS are putative, based on homology and distance from the RAE. The TSS and putative −35 and −10 consensus elements (σ^A^/σ^B^, (Zhu et al. 2017)) are highlighted with grey background. Conserved Gs (immediately upstream of −10, underlined) are typical for mycobacterial promoters (Zhu et al. 2017). The RAE is in the rectangle, the palindrome indicated with arrows. (**B**) Rifampicin can bind RNAP, RNAPσ^A^. Rifampicin blocks transcription at an early stage. Elongating RNAP can be stalled on DNA by the presence of an obstacle. (**C**) HelD can dissociate RNAP from nucleic acids and rifampicin. The light green part of HelD indicates that this part of HelD is obscured by RNAP. (**D**) Binding of ATP/GTP (ATP is shown only for simplicity) induces dissociation of HelD from RNAP. Liberated RNAP can re-enter the transcription cycle. (**E**) HelD hydrolyzes ATP/GTP and can interact with RNAP again. HelD is also generated by translation of helD mRNA, especially after induction of *helD* transcription by rifampicin. For more details, see text.

Regulation of Class II HelD expression may also depend on two promoters as in *M. smegmatis* (Martini et al. 2019) promoter 1 and 2 were identified to drive expression of the *helD* gene (Fig. 5A). The experimental evidence is available only in the absence of rifampicin when expression of HelD is relatively low, which is sufficient for its role in maintenance of genome integrity. Based on homology, the 5’ UTR of *M. abscessus helD* displays the same promoter architecture. The stimulation of expression of Class II HelD by rifampicin is controlled at the transcriptional rather than translational level (Hurst-Hess et al. 2019, Hurst-Hess et al. 2022). The RAE, required for the stimulation, is ∼20 bp downstream from the transcription start of the σ^A^/σ^B^-dependent promoter 1 and overlaps with the −35 region of the σ^A^/σ^B^-dependent promoter 2. The RAE consists of a 19 bp palindromic sequence (Spanogiannopoulos et al. 2014). To such *cis*-regulatory palindromes, transcription factors are known to bind (Narlikar and Hartemink 2006), either as homodimers or heterodimers, in the latter case offering combinatorial gene expression regulation.

When the upstream half of the RAE was deleted, abolishing the palindrome, the induction of *helD* by rifampicin was lost (Hurst-Hess et al. 2022). This loss of inducibility could be due to two reasons. First, the partial deletion of the RAE also removed the –35 region of promoter 2, likely compromising its activity. Second, association of an unknown, rifampicin-binding activator with the RAE was prevented.

In *B. subtilis*, the *helD* promoter activity is clearly inducible by rifampicin even though it does not contain the RAE (Hutter et al. 2004, Urban et al. 2007). On the other hand, the involvement of HelD in rifampicin resistance has not been detected and Class I HelDs lack the PCh loop (Surette et al. 2022). It is difficult to reconcile these results and we speculate that perhaps using more sensitive assays (*e.g*. time kill), it may turn out that *Bsu* HelD as well as other Class I and possibly also Class III HelDs are involved in rifampicin resistance albeit not as strongly as in *Actinobacteria*. The key function of the PCh loop in rifampicin resistance may in Class I HelDs instead be mediated by amino acid residues in the N-terminal domain that directly interact with amino acid residues of the rifampicin binding pocket (Pei et al. 2020). Fig. 4B illustrates this situation, showing the potential of Class I HelD to interfere with rifampicin binding. HelD forces part of β, most notably E521, into the rifampicin binding pocket.

Potentially, the δ subunit of RNAP may also be involved as its flexible C-terminal domain may extend into the DNA/RNA channel. Regardless of whether *Bsu* HelD has some role in rifampicin resistance or not, the experimentally demonstrated rifampicin-dependent induction of its expression is mediated by a yet to be determined mechanism.

## Model of the HelD cycle

The current knowledge maps many, but not all, of the steps of the HelD cycle in transcription and rifampicin resistance. Also, there are differences with respect to what types of RNAP complexes HelD binds between *B. subtilis* (Class I) and *M. smegmatis* (Class II). *Bsu* HelD was shown to bind the RNAP core (Wiedermannova et al. 2014) while *Msm* HelD was found to bind both the RNAP core and holoenzyme containing σ^A^ and RbpA (Kouba et al. 2020). RbpA is an *Actinobacteria*-specific transcription factor that increases the rate of open complex formation between RNAPσ^A^ and promoter DNA (Jensen et al. 2019). Interestingly, RbpA was previously reported to affect rifampicin resistance in *Actinobacteria* (Newell et al. 2006). This effect seems to stem from its ability to boost transcription in general and not from affecting rifampicin interaction with RNAP (Hu et al. 2012) although a study by Verma *et al*. reported that in an *in vitro* heterologous system *Mtu* RbpA was capable of rescuing rifampicin-induced transcription inhibition of *Msm* RNAP (Verma and Chatterji 2014).

Both Class I and II HelDs can bind to stalled elongation complexes and dissociate them (Kouba et al. 2020, Hurst-Hess et al. 2022). The binding likely occurs in a nucleotide free state and it is believed to be initiated by the N-terminal domain of HelD. Binding of ATP/GTP is required for dissociation of Class I HelD from RNAP (Pei et al. 2020). This effect of ATP/GTP binding on Class II HelD release from RNAP has not been tested yet. This dissociation may be further stimulated by interaction with DNA but experimental evidence is lacking. We speculate that in the cytoplasm, HelD then hydrolyzes ATP, resetting the molecule for another round of interactions. With respect to rifampicin, Class II HelD was shown to dissociate rifampicin-stalled RNAPs from DNA and also rifampicin from RNAP in the absence of nucleic acids. A simplified model of the Class II HelD cycle is depicted in Fig. 5B-E. We note that this model aggrees in most aspects with the model presented in (Hurst-Hess et al. 2022). The main difference is the mode of HelD release from RNAP with respect to ATP hydrolysis.

Finally, when ATP/GTP levels are relatively low in the cell, such as during nutritional starvation in stationary phase, the absence of ATP/GTP binding likely contributes to sequestration of RNAP by Class I HelD, keeping it in an inactive form until conditions improve (Pei et al. 2020). In the case of Class II HelDs, this scenario has not been explored.

## Concluding remarks

Bacterial HelD proteins (all Classes) interact in a unique manner with RNAP. Some aspects of this interaction are yet to be specified, such as the sequence of events leading to, and during HelD/HelR release from RNAP. Class II HelD (HelR) proteins then represent a new mode of rifampicin resistance. Interestingly, resistance to another RNAP-binding antibiotic fidaxomicin is not affected by Class II HelD proteins (Surette et al. 2022) even though HelD binding to RNAP dilates the RNA exit channel (Kouba et al. 2020)—the fidaxomicin binding site (Boyaci et al. 2018). Future studies will be required to elucidate the exact mechanism by which rifampicin is released by Class II HelDs from RNAP. It also remains to be explored in more detail whether Class I and III HelDs possibly also play some role in rifampicin resistance. Moreover, resistance to rifampicin might also be influenced by the presence of other interacting microbes (Bottery et al. 2021); deciphering such effects within complex communities may yield medicinally relevant insights. Last but not least, addressing the mechanisms of rifampicin-dependent expression control then is of prime interest and may reveal novel types of transcriptional regulation.

Finally, the discovery of rifampicin resistance by Class II HelD proteins reveals how relatively little we know about even well-studied antibiotics even though the effects of sub-MIC of antibiotics have been long recognized (Davies et al. 2006). ‘Omic’ approaches similar to those used previously with rifampicin (Giddey et al. 2017, Hurst-Hess et al. 2019, Surette et al. 2022) may be applied to other antibiotics when their sub-MIC may lead to upregulation of previously unidentified proteins involved in antibiotic resistance, and to a better understanding of risks posed by hormetic dose responses of bacteria to environmental contamination by antibiotics due to their overuse (Iavicoli et al. 2021).
